# The clinical challenge of refractory eosinophilic fasciitis

**DOI:** 10.1097/j.pbj.0000000000000230

**Published:** 2023-10-16

**Authors:** Daniela Oliveira, Ana Martins, Filipe Pinheiro, Maria Rato, Diogo Fonseca, Carlos Vaz, Pedro Madureira, Lúcia Costa

**Affiliations:** a Rheumatology Department, Centro Hospitalar Universitário São João, Porto, Portugal; b Center for Health Technology and Services Research (CINTESIS), Faculty of Medicine, University of Porto, Porto, Portugal; c Rheumatology Department, Centro Hospitalar Vila Nova de Gaia/Espinho, Gaia, Portugal; d Department of Medicine of Faculty of Medicine, University of Porto, Porto, Portugal

## To the Editor:

Eosinophilic fasciitis (EF) is a rare connective tissue disease, with unclear etiology, characterized by hardening and thickening of the skin, mainly affecting the upper and lower extremities. This condition is associated with peripheral eosinophilia, elevated erythrocyte sedimentation rate (ESR), and hypergammaglobulinemia. Most patients with EF respond to high-dose corticosteroids. Thus, a case of EF is being reported for its rarity and partial response to prednisolone.

**Figure 1.**
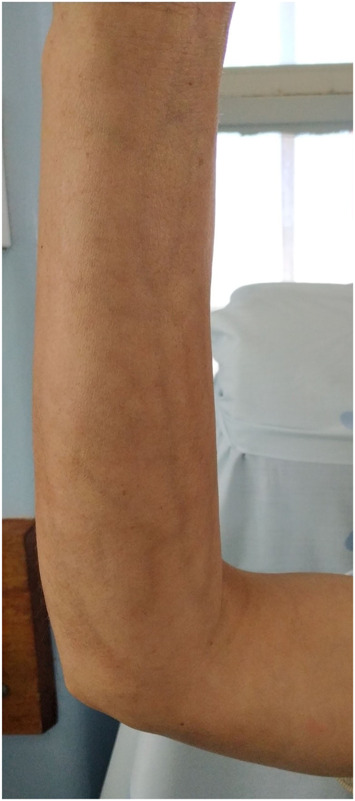
Groove sign.

We report a case of a 47-year-old woman with a personal history of multinodular goiter and no usual medication. This patient was admitted to the rheumatology service because of pain and skin hardening of right upper and lower limbs for the past 5 months. There were no systemic complaints; skin rash; Raynaud phenomenon; genital or oral ulcers; and respiratory, gastrointestinal, or genitourinary manifestations. No trauma or exacerbated physical activity was reported. During these months, the patient was medicated with an anti-inflammatory drug and low-dose corticosteroid for a small period, without significant relief. General physical examination was normal. On physical examination, skin thickening was observed on the right leg and forearm. On the forearm, the groove sign was visible when the patient raised the upper limb (Fig. 1). Left limbs, hands, and fingers were unaffected. There were no other mucocutaneous changes or peripheral arthritis.

On investigation, she had peripheral eosinophilia (0.8 × 10^9^/L, normal range, <0.5 × 10^9^/L), an elevated ESR (43 mm in the first hour), and proteinogram with a polyclonal hypergammaglobulinemia (gamma region). Hemogram, platelet, hepatic parameters, renal function, muscle enzymes, complement, and immunoglobulins were normal. She had a slightly positive rheumatoid factor (38 UI/mL, normal range <30 UI/mL) and a high titer antinuclear antibody (ANA)—1/640 with a homogeneous pattern. Remaining immunological studies, namely anti–citrullinated protein antibody (anti-CCP), extractable nuclear antigen antibodies (ENA), antineutrophil cytoplasmic antibodies (ANCA), and anti–double-stranded DNA (anti-dsDNA) antibodies, were negative. Osteoarticular radiographs (hands, elbows, knees, ankles) and ultrasounds of the right forearm and leg were normal. Magnetic resonance imaging (MRI) of the right forearm documented thickening, edema, and increased uptake of the fascial planes, without significant muscle involvement. Deep skin and muscle biopsy showed fibrinous exudate, marked lymphoplasmacytic inflammatory infiltrate, and occasional eosinophils, findings compatible with EF. A solid, hypoechoic nodule was found on thyroid ultrasound, and an aspiration biopsy concluded it to be a benign colloid nodule. Additional investigation with abdominopelvic ultrasound, breast mammography, and cervicovaginal cytology showed no pathological findings.

Prednisolone 1 mg/kg/day treatment was started with partial clinical and analytical response. Methotrexate was initiated early and prednisolone was slowly tappered, with favorable pain response and sustained decline of peripheral eosinophilia and ESR. After 2 years of follow-up in rheumatology consultation, under methotrexate 25 mg/week, the patient is significantly better, with residual skin thickening and hardening, mainly on the forearms. Follow-up MRI of the right forearm showed a slight thickening and increased uptake without other alterations.

EF is a rare clinical condition with delayed diagnosis. A high index of clinical suspicion of EF is needed to establish an early diagnosis and to target treatment preventing unnecessary invasive examinations. Treatment of EF remains poorly understood with no published clinical guidelines. Although most cases respond well to high doses of oral corticosteroids, some patients may require a steroid-sparing agent, the most common being methotrexate. However, for refractory cases, there are no approved therapeutic options. Clinical trials are needed to explore the long-term safety and effectiveness of other immunosuppressive agents in refractory EF.

## Conflicts of interest

The authors declare that they have no conflicts of interest.

